# X-ray Image Enhancement Based on Nonsubsampled Shearlet Transform and Gradient Domain Guided Filtering

**DOI:** 10.3390/s22114074

**Published:** 2022-05-27

**Authors:** Tao Zhao, Si-Xiang Zhang

**Affiliations:** 1 School of Mechanical Engineering, Hebei University of Technology, Tianjin 300131, China; 92zhaotao@163.com; 2 Department of Mechanical Engineering, Zhonghuan Information College Tianjin University of Technology, Tianjin 300380, China

**Keywords:** X-ray image, image enhancement, non-subsampled shearlet transform, adaptive gamma correction with weighting distribution, gradient-domain guided filtering

## Abstract

In this paper, we propose an image enhancement algorithm combining non-subsampled shearlet transform and gradient-domain guided filtering to address the problems of low resolution, noise amplification, missing details, and weak edge gradient retention in the X-ray image enhancement process. First, we decompose histogram equalization and nonsubsampled shearlet transform to the original image. We get a low-frequency sub-band and several high-frequency sub-bands. Adaptive gamma correction with weighting distribution is used for the low-frequency sub-band to highlight image contour information and improve the overall contrast of the image. The gradient-domain guided filtering is conducted for the high-frequency sub-bands to suppress image noise and highlight detail and edge information. Finally, we reconstruct all the effectively processed sub-bands by the inverse non-subsampled shearlet transform and obtain the final enhanced image. The experimental results show that the proposed algorithm has good results in X-ray image enhancement, and its objective index also has evident advantages over some classical algorithms.

## 1. Introduction

X-ray image has become widely employed in medical diagnosis, security inspection, aerospace, defect detection, machinery manufacture, and other industries since the development of photoelectric detecting technology and image analysis technology. The radiographic inspection system’s detecting principle, the image of the hardware equipment, and the picture created by the X-ray instrument all suffer from low dynamic range, low definition, low contrast, and excessive noise. Defect detection and image analysis are performed directly on the collected image. It causes significant inaccuracy in the detection results. As a result, it is beneficial to use image enhancement algorithms to analyze X-ray images [1,2], increase image quality and visual effects, and make subsequent detection easier.

Spatial domain pixel enhancement and transform domain multi-scale coefficient improvement enhancement methods are the most common image enhancement algorithms. The spatial domain improvement is to improve the image by directly processing the pixels, such as histogram equalization [3,4,5], image sharpening and grayscale stretching [6,7,8], and retinex theory [9]. Zeng et al. [10] proposed a gray-level information histogram for X-ray image contrast enhancement, which improves the performance of many histogram-based enhancement techniques dramatically. However, when the image is enhanced, there will be an over-enhancement phenomenon that will cause the image to be distorted. Panetta et al. [11] introduced nonlinear unsharp masking for mammogram enhancement. This method has good performance for enhancing the fine details in the original images. However, it also amplifies noise and overshoots the sharp details at the same time. Tao et al. [12] introduced a retinex-based framework for medical X-ray image enhancement. The framework can increase the contrast, eliminate the noise, and enhance the details, but the contour edges of the image are affected by the dark area pixels in the original image, showing shadows that did not exist at all.

The enhancement based on the transform domain first transforms the original image into the frequency domain for multi-scale decomposition, amplifies or filters the decomposed sub-image, and then inversely transforms the image. The wavelet transform [13], ridgelet transform [14], curvelet transform [15], wedgelet transform [16], contourlet transform [17], nonsubsampled contourlet transform(NSCT) [18,19], shearlet transform [20,21], nonsubsampled shearlet transform (NSST) [22], and other transformations are commonly used. Tang et al. [23] proposed an algorithm based on a multiscale measure in the wavelet domain for screening mammograms to enhance the details at different scales. However, the wavelet transform can only capture information in three directions: horizontal, vertical, and diagonal, which is poor in representing anisotropic singular features in images. Ostojié et al. [24] proposed an intensity adaptive nonlinear multiscale detail and contrast enhancement algorithm for digital radiography. The method adapts to the local exposure level and thus reduces the artifacts’ saliency, but the adaptation of detail enhancement to image pixel intensity needs to be strengthened. Zhou et al. [25] introduced a medical image enhancement method based on improved gamma correction in the shearlet domain, which makes the texture details of the image more prominent and the overall contrast is significantly improved. Nevertheless, since this method does not have translation invariance, the image will produce a pseudo-Gibbs phenomenon.

Because of the unconstrained shearing directions, images after NSST may achieve optimal sparse representation and nonlinear error approximation. There have been many achievements in applying the NSST to image enhancement. Zhang et al. [26] employed NSST and tetrolet transform to remote sensing images, effectively retaining the details and edges of the image and significantly improving the information entropy and mean; Li et al. [27] studied the NSST domain to improve blur. The contrast enhancement algorithm uses the remote sensing image enhancement coefficient as an adjustable pattern recognition task, effectively removing the pseudo-Gibbs phenomenon from the image during the enhancement process. Tong et al. [28] proposed a visual sensor image enhancement algorithm using NSST and phase stretching transformation. In this method, the author uses nonlinear models with different thresholds to process the different scale parts after NSST decomposition. The algorithm can suppress noise and effectively increase the contrast of the image. However, none of the above studies have analyzed the parameters and effects of the decomposition levels and shearing directions of NSST, and the parameter selection has a certain degree of randomness. 

He et al. [29] proposed a linear edge-preserving guided image filtering algorithm. The filtered image can avoid a blurring effect on detailed information. Li et al. [30] introduced a weighted guided image filter by incorporating an edge-aware weighting into an existing guided image filter to address the problem of abiding by halo artifacts. Kou et al. [31] proposed gradient-domain guided filtering to reduce the effect of image edge smoothing and introduced first-order edge-aware constraints to processing images, which can better preserve image edges. However, using intensity domain constraints for edges and details can over-smooth edges and reduce edge retention.

To address the above issues, we propose an image enhancement method. It is based on NSST and gradient-domain guided filtering and applies it to X-ray images. The algorithm combines the advantages of NSST for sparse image representation with gradient-domain guided filtering for image detail enhancement. The low-frequency sub-band uses adaptive gamma correction with a weighted distribution to enhance contrast, highlighting tiny details in the background. The high-frequency sub-bands use gradient-domain guided filtering to filter out image noise and extract edge and texture information by analyzing the image quality of the four-levels direction decomposition under different scale decomposition levels, and the image quality of various direction decomposition sequences under the four-levels scale decomposition. We compare the running times of different decompositions and obtain optimal NSST decomposition parameters. The enhancement experiments on medical and industrial X-ray images show that the proposed algorithm can enhance the image contrast, details, and texture information and obtain high-quality images for subsequent research and analysis.

## 2. Related Works

### 2.1. Nonsubsampled Shearlet Transform

The nonsubsampled shearlet transform is extended based on the shearlet transform. The shearlet transform is an algorithm that combines synthetic dilated affine systems with multiscale analysis. It can decompose the image more sparsely and achieve optimal approximation. Its construction is simple and anisotropic, and in dimension n=2, the affine systems with composite dilations are collections of the form:

(1) $$\Lambda_{AB}(\psi )=\left\{\psi_{j,l,k}(x)={\left|\det A\right|}^{j/2}\psi (B^{l}A^{j}x-k):j,l\in \mathbb{Z},k\in {\mathbb{Z}}^{2}\right\}$$

(2) $$A=\left(\begin{array}{cc} a & 0\\ 0 & \sqrt{a} \end{array}\right),\;B=\left(\begin{array}{cc} 1 & s\\ 0 & 1 \end{array}\right)$$

Among them, $\psi \in L^{2}({\mathbb{R}}^{2})$ represents the basis function, A is a 2-dimensional invertible matrix of anisotropic expansion, which is related to scale transformation. B is a 2-dimensional shear invertible matrix, related to rotation or shear transformation, and |detB|=1, j,l,k represents the scale parameter, shearing parameter, and translation parameter. If the system $\Lambda_{AB}(\psi )$ forms a Parseval frame (also called a tight frame) for $L^{2}({\mathbb{R}}^{2})$, then ψ in the system is called a synthetic wavelet, and the following formula holds for all $f\in L^{2}({\mathbb{R}}^{2})$:

(3) $${\sum_{j,l,k}\left|\langle f,\psi_{j,l,k}\rangle \right|}^{2}={\left\|f\right\|}^{2}$$

**Figure 1:**
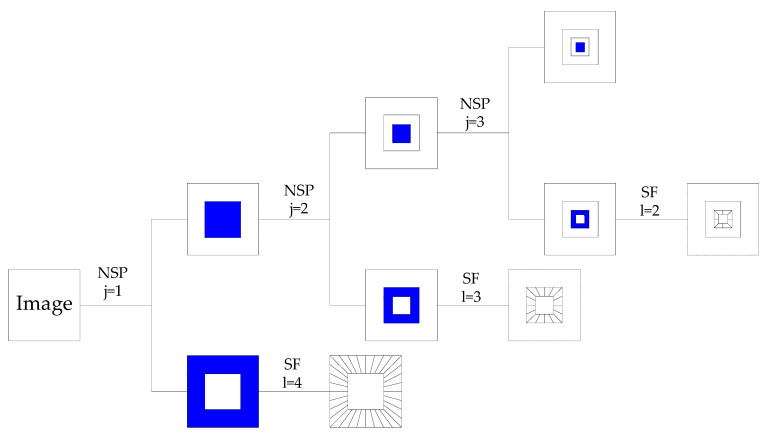
NSST of the three-levels decomposition process.

This synthetic wavelet is called a shear wave when a=4,s=1. As shown in Figure 1, the three-levels NSST decomposition structure diagram, in which the scale parameter j=3 and the numbers of the shearing parameters of each level are set to [2,3,4] respectively, then the corresponding shearing directions of each level are [4,8,16].

### 2.2. Adaptive Gamma Correction with Weighted Distribution

Gamma correction can expand the bright part in the X-ray image to make the image contrast more obvious, and the simple form of the transform-based gamma correction is derived by

(4) $$T(v)=v_{\max}{\left(v/v_{\max}\right)}^{\gamma }$$

where v is the grayscale of the input image and $v_{\max}$ is the maximum intensity of the input image, and γ is the varying adaptive parameter. 

Define the probability density function of each gray level in the image as pdf approximated by

(5) $$pdf(v)=n_{v}/(MN)$$

where $n_{v}$ is the number of pixels whose gray level is v, MN is the total number of image pixels, and its cumulative distribution function cdf is defined as

(6) $$cdf(v)=\sum_{k=0}^{v}pdf(v)$$

The weighted distribution function is defined as

(7) $$pdf_{\omega }(v)=pdf_{\max}{\left(\frac{pdf(v)-pdf_{\min}}{pdf_{\max}-pdf_{\min}}\right)}^{\alpha }$$

Adjust the histogram of statistics with a weighted distribution function, where α is the adjustment parameter, $pdf_{\max}$ is the maximum pdf of the statistical histogram, and $pdf_{\min}$ is the minimum of the statistical histogram. Applying cdf(v) and $pdf_{\omega }(v)$ to the normalized gamma function, the formula of adaptive gamma correction with weighting distribution (AGCWD) [32] is obtained as

(8) $$T(v)=v_{\max}{\left(v/v_{\max}\right)}^{\gamma_{A}}$$

where

(9) $$\gamma_{A}=1-\sum_{v=0}^{v_{\max}}pdf_{\omega }(v)/\Sigma pdf_{\omega }$$

with

(10) $$\Sigma pdf_{\omega }=\sum_{v=0}^{v_{\max}}pdf_{\omega }(v)$$

Since most of the pixels of the X-ray image are densely distributed in the low grayscale area, the AGCWD algorithm can gradually increase the low pixel intensity of the image based on the weight distribution function, smooth the fluctuation phenomenon, and thus reduce the excessive enhancement of the image by gamma correction.

### 2.3. Gradient Domain Guided Filtering

The output of any pixel in the filtered image can be expressed as the following linear model:

(11) $$\overset{\wedge }{Z}(p)=a_{p^{\prime }}G(p)+b_{p^{\prime }},\forall p\in \Omega_{\varsigma_{1}}(p^{\prime })$$

Among them, $\overset{\wedge }{Z}(p)$ is the output image, G(p) is the guide image, $\Omega_{\varsigma_{1}}(p^{\prime })$ is a local square window with the point $p^{\prime }$ as the center and $\varsigma_{1}$ as the radius in the guide image G(p), $a_{p^{\prime }}$ and $b_{p^{\prime }}$ are the constant term coefficients in the window $\Omega_{\varsigma_{1}}(p^{\prime })$. To compute $a_{p^{\prime }}$ and $b_{p^{\prime }}$, we define $E(a_{p^{\prime }},b_{p^{\prime }})$ (hereafter abbreviated as E) as the noise-dependent loss function within the window $\Omega_{\varsigma_{1}}(p^{\prime })$ as follows:

(12) $$E=\sum_{p\in \Omega_{\varsigma_{1}}(p^{\prime })}[(a_{p^{\prime }}G(p)+b_{p^{\prime }}-X(p){)}^{2}+\frac{\lambda }{{\overset{\wedge }{\Gamma }}_{G(p^{\prime })}}{\left(a_{p^{\prime }}-\gamma_{p^{\prime }}\right)}^{2}]$$

Among them, X(p) is the image to be filtered, λ is the regularization parameter to prevent $a_{p^{\prime }}$ from being too large, and ${\overset{\wedge }{\Gamma }}_{G(p^{\prime })}$ is the edge perception weight, which is defined as follows:

(13) $${\overset{\wedge }{\Gamma }}_{G(p^{\prime })}=\frac{1}{N}\sum_{p=1}^{N}\frac{\chi (p^{\prime })+\varepsilon }{\chi (p)+\varepsilon }$$

(14) $$\chi (p^{\prime })=\sigma_{G,1}(p^{\prime })\times \sigma_{G,\varsigma_{1}}(p^{\prime })$$

where $\sigma_{G,1}(p^{\prime })$ and $\sigma_{G,\varsigma_{1}}(p^{\prime })$ represent the standard deviation within the window 3×3 and within the window (2ς+1)×(2ς+1), centered on the point $p^{\prime }$. ε defined as ${\left(0.001\times L\right)}^{2}$, L is the dynamic range of the input image. $\gamma_{p^{\prime }}$ is the edge image factor, defined as follows:

(15) $$\gamma_{p^{\prime }}=1-\frac{1}{1+e^{\eta (\chi (p^{\prime })-\mu_{\chi ,\propto })}}$$

where $\mu_{\chi ,\propto }$ is the mean of χ(p), and η is calculated as $4/(\mu_{\chi ,\propto }-\min(\chi (p)))$. It can be known from formula (15) that if the pixel $p^{\prime }$ is in the smooth area of the image, the value of $\gamma_{p^{\prime }}$ is close to 0, and if it is at the edge of the image, the value of $\gamma_{p^{\prime }}$ is close to 1.

To minimize the noise of the filtered image, take the minimum value of E, and the linear regression is used to solve the formula (12) to obtain

(16) $$a_{p^{\prime }}=\frac{\mu_{G⊙X,\varsigma_{1}}(p^{\prime })-\mu_{G,\varsigma_{1}}(p^{\prime })\mu_{X,\varsigma_{1}}(p^{\prime })+\frac{\lambda }{{\overset{\wedge }{\Gamma }}_{G(p^{\prime })}}\gamma_{p^{\prime }}}{\sigma_{G,\varsigma_{1}}^{2}(p^{\prime })+\frac{\lambda }{{\overset{\wedge }{\Gamma }}_{G(p^{\prime })}}}$$

(17) $$b_{p^{\prime }}=\mu_{X,\varsigma_{1}}(p^{\prime })-a_{p^{\prime }}\mu_{G,\varsigma_{1}}(p^{\prime })$$

where ⊙ is the dot product between the two matrices,$\mu_{G⊙X,\varsigma_{1}}(p^{\prime })$, $\mu_{G,\varsigma_{1}}(p^{\prime })$ and $\mu_{X,\varsigma_{1}}(p^{\prime })$ are the mean values of G⊙X, G, and X. Bringing formulas (16) and (17) into formula (11), the final calculation formula of $\overset{\wedge }{Z}(p)$ is simplified as

(18) $$\overset{\wedge }{Z}(p)={\overset{-}{a}}_{p}G(p)+{\overset{-}{b}}_{p}$$

where ${\overset{-}{a}}_{p}$ and ${\overset{-}{b}}_{p}$ are the mean values of $a_{p^{\prime }}$ and $b_{p^{\prime }}$ in the window $\Omega_{\varsigma_{1}}(p)$.

Gradient-domain guided filtering preserves its detailed features while smoothing the image. To further enhance the edge and texture information of the image, the smoothed image is subtracted from the original image to obtain a different image, which is added to the smoothed image to obtain an enhanced one, and the specific formula is as follows

(19) $$G_{enhanced}=\overset{\wedge }{Z}(p)+\xi (X(p)-\overset{\wedge }{Z}(p))$$

where ξ is the scale coefficient of the differential gain effect of the image gradient-domain guided filtering.

## 3. Implementation of the Algorithm

### 3.1. Algorithm Implementation Steps

**Figure 2:**
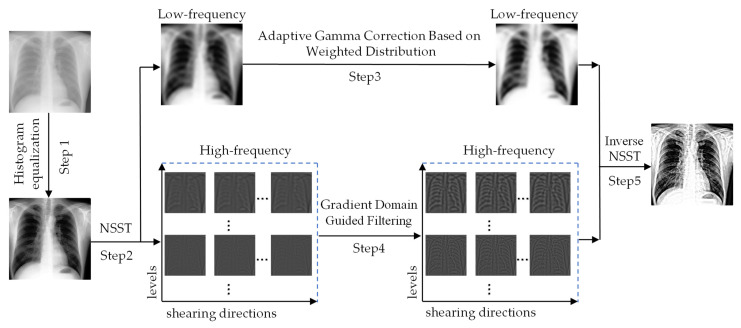
The proposed method for enhancing X-ray images is depicted schematically.

The flow of the enhancement algorithm is shown in Figure 2. The high-frequency sub-bands of the image contain noise, and as the decomposition scale increases, they become almost invisible. We use gradient-domain guided filtering to process the high-frequency sub-bands to reduce noise interference. The detailed information in the image can be well preserved. To display the high-frequency images more clearly, both the high-frequency sub-bands and the images enhanced by the gradient domain guided filtering have undergone a linear grayscale transformation. 

Step 1: Perform histogram equalization on the X-ray image, stretch the overall grayscale range of the image, and improve the image layering.

Step 2: Perform NSST scale decomposition on the image processed in Step 1 to obtain one low-frequency sub-band and multiple high-frequency sub-bands.

Step 3: Use adaptive gamma correction with weighted distribution to enhance the contrast of the low-frequency sub-band to highlight a small amount of detailed information in the background.

Step 4: Use gradient-domain guided filtering for the high-frequency sub-bands to filter out image noise and subtract the smoothed image from the original image to obtain a differential image, which was added to the smoothed image by scale coefficient ξ to perform image enhancement.

Step 5: Perform inverse NSST on the processed low-frequency sub-band and high-frequency sub-bands and output the final enhanced image.

### 3.2. Decomposition Levels Analysis of NSST

The key parameters of NSST decomposition are the decomposition levels and shearing directions of each level. To analyze the effect of decomposition levels on image quality, five X-ray images of different sizes and different grayscales were selected for the experiments. Experiments were performed on the 64-bit operating system of Windows 10 (Intel Core i7-8750H CPU2.20GHz), and the experimental tool was MATLAB R2016b. The scale decomposition levels are set to 1-5, respectively, the number of shearing parameter of each level is set to 4, and the corresponding shearing directions of each level are 16. The window radius of the gradient domain guided filtering ς is 16, the regularization parameter λ is 0.5, the scale coefficient ξ is 5, and the parameters of other experimental conditions are kept the same.

**Figure 3:**
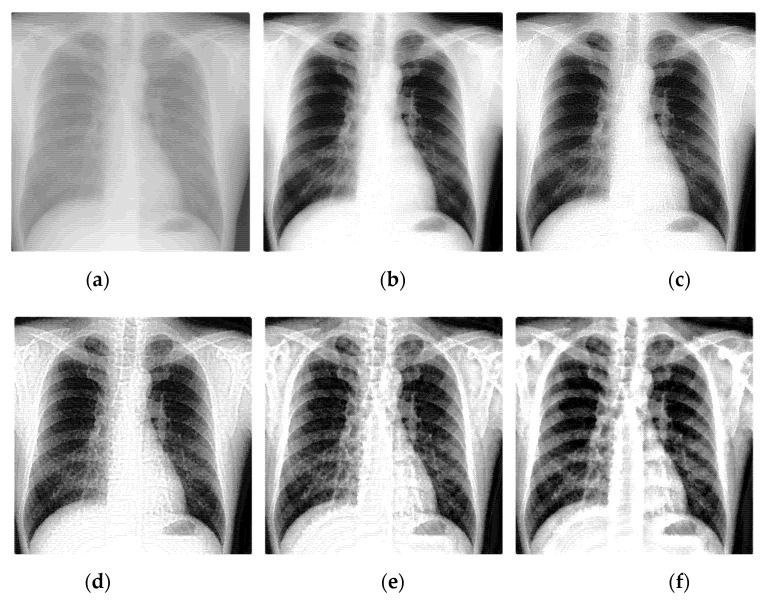
The enhanced effects of different decomposition levels on the X-ray image 1. (**a**) is the original image, and (**b**–**f**) corresponds to the decomposition levels j equal to 1–5.

**Figure 4:**
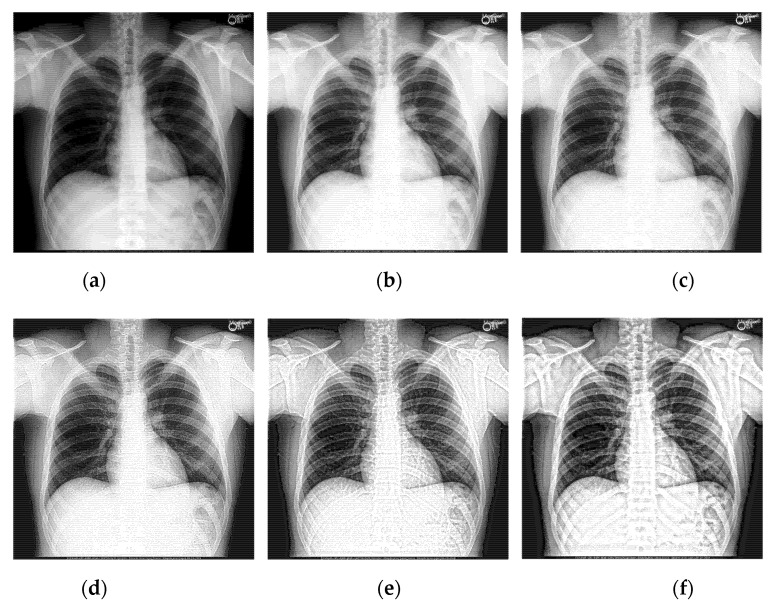
The enhanced effects of different decomposition levels on the X-ray image 2. (**a**) is the original image, and (**b**–**f**) corresponds to the decomposition levels j equal to 1–5.

Select two representative medical images for analysis: Image 1 with the size of 440×440 and image 2 with the size of 1024×1024. The enhanced X-ray images obtained under different decomposition levels are shown in Figure 3 and Figure 4 where (a) is the original image, and (b–f) corresponds to the decomposition levels j equal to 1–5. When the number of scale decomposition levels is j, NSST decomposition requires 2j times of image and filter convolution; the running time of the algorithm gradually increases. Observing the image, the image contrast has been significantly improved after histogram equalization. When the decomposition scale j≤3, with the increase of the NSST decomposition scale, the boundary and texture features of the image are gradually obvious, and the detailed information is enhanced. When 5≥j>3, the enhancement effect is further improved; the change is not significant.

The subjective evaluation of the enhancement effect purely from the visual aspect has a certain one-sidedness. Therefore, five indicators of average gradient (AG), information entropy (H), spatial frequency (SF), edge intensity (EI), and running time (RT) are selected to objectively analyze the enhancement effect.

The average gradient (AG) can reflect the sharpness of the image and is defined as:

(20) $$AG=\frac{\sum_{i}\sum_{j}{\left({\left(f(i,j)-f(i+1,j)\right)}^{2}+{\left(f(i,j)-f(i,j+1)\right)}^{2}\right)}^{\frac{1}{2}}}{MN}$$

The larger the average gradient of an image, the higher the image clarity.

The information entropy (H) is an important indicator to measure the richness of image information and is defined as:

(21) $$H(p)=\sum_{l=0}^{L-1}P(l)\log P(l)$$

where L refers to the number of gray levels, and P(l) represents the distribution probability of each gray level. The information entropy value indicates the average amount of information contained in the enhanced image. The larger the value, the richer the information contained in the enhanced image.

Spatial frequency (SF) can reflect the overall activity of an image in the spatial domain. The higher the spatial frequency, the better the quality of the enhanced image. Its formula is defined as follows:

(22) $$SF=\sqrt{RF^{2}+CF^{2}}$$

Among them, RF represents the spatial row frequency and CF represents the spatial column frequency; the definitions of RF and CF are as follows:

(23) $$RF=\sqrt{\frac{1}{MN}\sum_{i=1}^{M}\sum_{j=2}^{N}{\left[f_{i,j}-f_{i,j-1}\right]}^{2}}$$

(24) $$CF=\sqrt{\frac{1}{MN}\sum_{j=1}^{N}\sum_{i=2}^{M}{\left[f_{i,j}-f_{i-1,j}\right]}^{2}}$$

The edge intensity (EI) reflects the image clarity degree. The more abundant the image detail and edge, the higher the image clarity.

(25) $$EI=\sum_{i}\sum_{j}\sqrt{G_{x}^{2}(i,j)+G_{y}^{2}(i,j)}$$

where $G_{x}(i,j)$, $G_{y}(i,j)$ represent the first-order partial derivative in horizontal and vertical directions and is defined as:

(26) $$G_{x}(i,j)=f(i,j)⊗g_{x}$$

(27) $$G_{y}(i,j)=f(i,j)⊗g_{y}$$

where ⊗ is the convolution symbol, $g_{x}$ and $g_{y}$ are the horizontal template and vertical template for the Sobel operator. When the kernel size is 3, they are defined as:

(28) $$g_{x}=\frac{1}{4}\left[\begin{array}{ccc} -1 & 0 & 1\\ -2 & 0 & 2\\ -1 & 0 & 1 \end{array}\right],\;g_{y}=\frac{1}{4}\left[\begin{array}{ccc} -1 & -2 & -1\\ 0 & 0 & 0\\ 1 & 2 & 1 \end{array}\right]$$

Having analyzed the effects of the decomposition levels on image enhancement, the statistical data is shown in Table 1 and Table 2.

**Figure 5:**
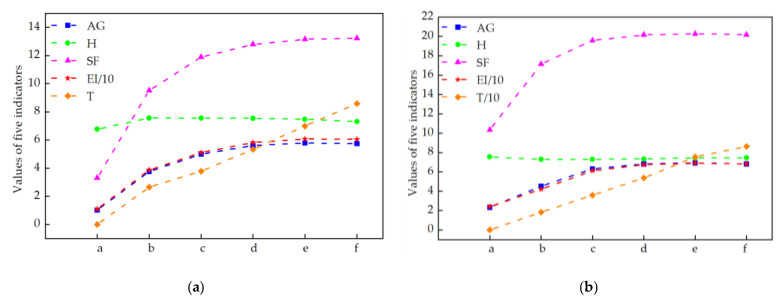
The effects of different decomposition levels: (**a**) are for Table 1 and (**b**) are for Table 2.

The larger the above four parameters are, the better the enhancement effect. Observing the data in Table 1 and Table 2 and Figure 5, under the condition of a certain number of shearing parameter in each level, with the increase of the decomposition scale, the information entropy slightly increases, but the overall fluctuation effect is not large. The average gradient, spatial frequency, and edge intensity of the image increase significantly, reaching their extreme value when the decomposition scale is 4. If the decomposition scale is increased again, the image enhancement effect is not obvious. Therefore, the primary decomposition scale is set to 4.

### 3.3. Shearing Directions Analysis of NSST

**Figure 6:**
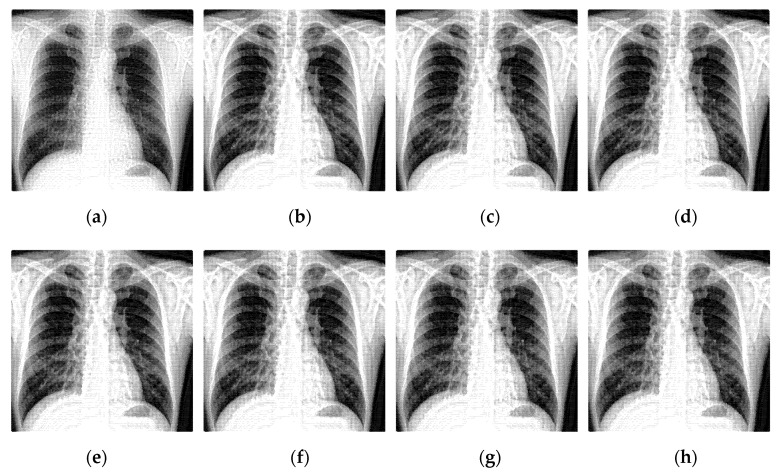
The enhanced effects of different shearing directions on X-ray image 1. In (**a**), the shearing parameters of each level are set to (4,4,4) and the shearing directions are (16,16,16). For (**b**–**h**), the decomposition scale was set to 4 levels, the shearing parameters of each level are set from (2,2,2,2) to (4,4,4,4), and the shearing directions are from (4,4,4,4) to (16,16,16,16). The number of shearing directions of each level was gradually increased.

**Figure 7:**
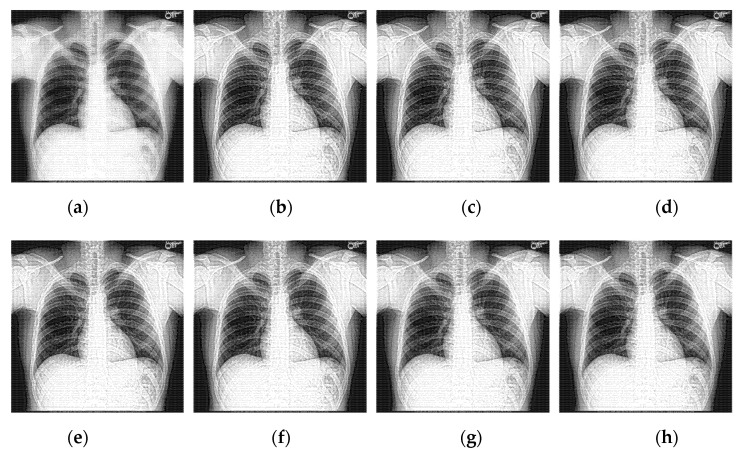
The enhanced effects of different shearing directions on X-ray image 2. In (**a**), the shearing parameters of each level are set to (4,4,4) and the shearing directions are (16,16,16). For (**b**–**h**), the decomposition scale was set to 4 levels, the shearing parameters of each level are set from (2,2,2,2) to (4,4,4,4), and the shearing directions are from (4,4,4,4) to (16,16,16,16). The number of shearing directions of each level was gradually increased.

Performing l-level directional decomposition on the high-frequency sub-band image can obtain a $2^{l}$-directional sub-band image of the same size as the source image, achieving more accurate directional decomposition in the frequency domain. The time taken by the 4-level directional decomposition of the NSST algorithm is much longer than that of the 2-level directional one. The shearing directions experiment was carried out on the same batch of pictures by selecting two representative medical images for analysis. The enhanced X-ray images obtained under different shearing directions are shown in Figure 6 and Figure 7. Having analyzed the effects of the shearing directions on image enhancement, the statistical data is shown in Table 3 and Table 4.

**Figure 8:**
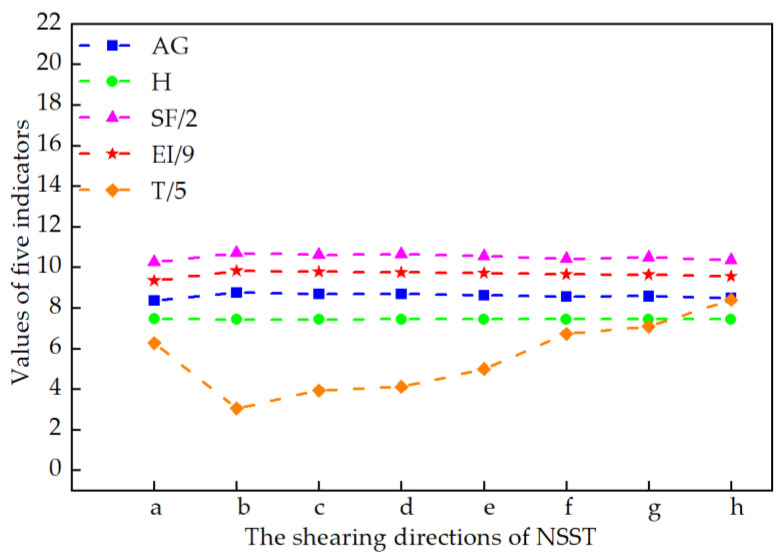
The effects of different shearing directions for Table 5.

To prove the effectiveness of the proposed algorithm in this paper, 30 chest X-ray images obtained from [33] are selected to simulate, and the average metrics are shown in Table 5. Five indicators of average gradient (AG), information entropy (H), spatial frequency (SF), edge intensity (EI), and running time (RT) are introduced to objectively analyze the enhancement effect. The line charts of the average values of the objective metrics data in Table 5 are given in Figure 8. To display the data in the same graph, the value of EI has taken one-ninth of the original value, the value of SF has taken half of the original value, and the value of T has taken one-fifth of the original value.

Observing the experimental data, under the condition of a certain decomposition scale, with the increase of the shearing parameter of each level, the operation time gradually increases due to the low efficiency of the iterative filtering operation during the direction division process. However, the average gradient, information entropy, spatial frequency, and edge intensity are not obvious, indicating that the number of clipping directions will increase the complexity of the operation and make the running time of the algorithm longer, but the enhancement effect on the image is not obvious. In the final algorithm scheme, the decomposition scale of NSST is set to four levels, the shearing parameters of each level are set to (2,2,2,2), and the shearing directions are (4,4,4,4).

## 4. Experimental Results and Discussion

### 4.1. Subjective Analysis

**Figure 9:**
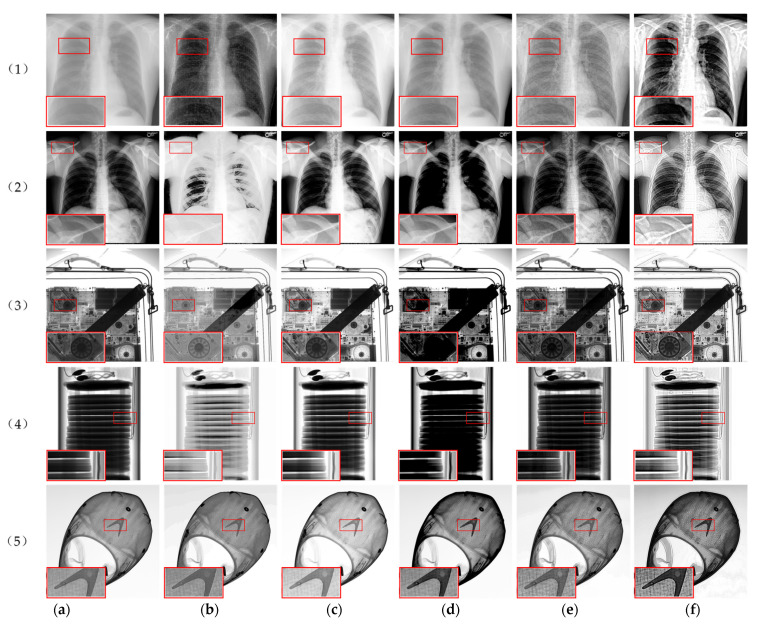
Comparison of different enhancement algorithms. (**a**) Original image, (**b**) FLM, (**c**) AGCWD, (**d**) TSSR, (**e**) LCM-CLAHE, (**f**) Proposed method.

To demonstrate the effectiveness of the algorithm in this paper, experiments were carried out on five X-ray images, as is shown in Figure 9, in which (1) and (2) are medical images mentioned before, (3) and (5) are industrial images with a size of 2048 × 2048, and (4) is a thermal battery image with a size of 1000 × 1000. The enhancement effect of the algorithm in this paper is compared with the FLM method [34], the AGCWD method [32], the TSSR method [9], and the LCM-CLAHE method [8] in terms of subjective visual effects and objective evaluation indicators. 

To facilitate the observation and analysis of the detailed information in the image, we zoomed in on part of the image, as shown in the red box in the experimental results. It can be seen from Figure 9 that the FLM method may appear excessively enhanced, such as the bone background in (b)(2) being too bright, and the single-cell stack of the battery in (b)(4) being too bright, making the image lose a large amount of detail information; the AGCWD method has a certain enhancement effect on the image clarity, but the enhancement of details and texture information is not obvious in (c)(1) and (2); the TSSR algorithm enhances the contrast, but it will make the black areas in the image connect to produce a blurring effect, such as (d)(3)–(5); the LCM-CLAHE method can improve the texture information of the image, such as (e)(1), (2) and (5), but because the image is dark and the contrast is low, it is not conducive to the observation of image details. Figure 9f shows the results of the algorithm used in this paper, which suggests that the method proposed in this paper is very effective for X-ray image enhancement. Dealing with medical X-ray images like Figure 9((1),(2)), we can see that the sharpness of bone and soft tissue information is significantly increased, image noise is suppressed, and contrast in local areas is also improved. The algorithm makes the texture information more prominent, which is beneficial to the doctor’s diagnosis and follow-up treatment of the patient’s disease. When applied to the industrial X-ray images in Figure 9((3)–(5)), we can see that the local details in the enhanced image are clearly visible, and the contrast between the battery texture information and the component edge information is obvious. The overall brightness of the image is moderate, and the noise components in the image are not seriously amplified so that the processed image is more in line with the visual effect of the human eye.

### 4.2. Objective Analysis

Four evaluation indicators, average gradient (AG), information entropy (H), spatial frequency (SF), and edge intensity (EI), were selected to objectively analyze the image enhancement effect. From the aggregated data in Table 6, we can see that the algorithm proposed in this paper has achieved the optimal values in the three indicators of average gradient, spatial frequency, and edge intensity compared with the other four enhancement algorithms, and the information entropy also ranks in second place. From the average index data of the five pictures, we can see that the proposed method has achieved the best results in all four indicators. 

The algorithm in this paper can improve the local contrast and sharpness, making it easier for people to obtain useful information about the target area from the enhanced image. To verify the robustness and general adaptability of the algorithm, experimental statistics were performed on 30 COVID-19 X-ray images with a size of 1024 × 1024 collected from [33]. The average metrics of their objective evaluation are given in Table 7. 

**Figure 10:**
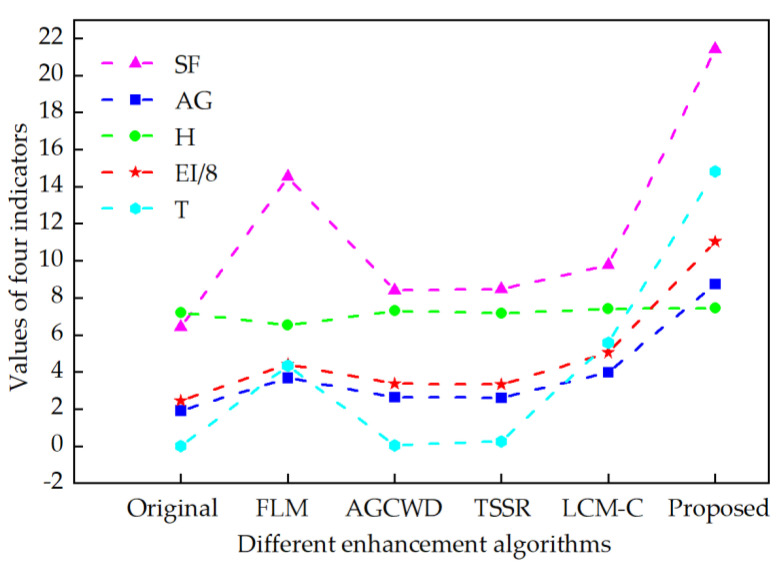
The average index of different enhancement algorithms on the 30 chest X-ray images was calculated.

The line charts of the average values of the objective metrics data in Table 7 are given in Figure 10. To display the data in the same graph, the value of EI has taken one-eighth of the original value. It can be seen from Figure 10 that the four evaluation indicators (AG, H, SF, and EI) obtained by the algorithm in this paper have achieved the optimal values among all the five algorithms. These results indicate that the proposed X-ray image enhancement algorithm can achieve a better enhancement effect. 

Compared with existing enhancement methods, the proposed algorithm does not show significant advantages in terms of running time. The image enhancement model based on shearlet transform has relatively high computational complexity. The choice of the number of decomposition layers influences the effect of image enhancement, and the increase in the number of decomposition layers will bring higher computational complexity. This results in a long time for image enhancement. The different processing of sub-bands after multi-scale decomposition also affects the image enhancement effect and running time. Therefore, how to select a better sub-band processing method to obtain the optimal enhancement effect while reducing the number of decomposition layers is the next problem we will study. In addition, some parameter values in the algorithm need to be determined by empirical values. For X-ray images of different initial conditions, the algorithm parameters need to be changed accordingly. Future research will focus on the adaptability of this method.

## 5. Conclusions

Due to the influence of the X-ray detection hardware system and other factors, the quality of the X-ray image will decline, and the visual effect will become worse, causing certain difficulties in the process of X-ray image detection. Therefore, it is necessary to perform enhancement processing on the radiographic image to obtain higher quality images for subsequent analysis. This paper proposes a new image enhancement algorithm combining NSST and gradient-domain guided filtering. The innovation is that the image is decomposed in the NSST domain. AGCWD is used to enhance the contrast of the low frequency. Gradient-domain guided filtering is performed on the high-frequency sub-bands to improve image details and texture features. The final enhanced image is obtained through NSST coefficient reconstruction. The algorithm makes full use of the image sparse representation characteristics of NSST, the advantages of adaptive gamma correction in image contrast enhancement, and the advantages of the gradient domain guided filter in image detail enhancement. The algorithm proposed in this paper has an obvious effect on image enhancement, not only improving the contrast and clarity of the image but also improving the image quality, as demonstrated by experiments on 30 X-ray images compared to the other four advanced image enhancement methods at home and abroad. From the experimental results, we can see that the evaluation indicators AG, H, SF, and EI have all achieved optimal value. This demonstrates that the proposed algorithm can obtain a better enhancement effect for both medical and industrial radiographic images. It will provide a basis for later medical X-ray image diagnosis and industrial X-ray defect identification and detection.

## Figures and Tables

**Table 1:** An objective evaluation of the NSST decomposition levels for Figure 3.

Figure	Levels (j)	$\mathrm{Directions}\;(2^{l})$	AG	H	SF	EI	RT
Figure 3a	/	/	1.0209	6.7705	3.2987	11.0071	0
Figure 3b	1	16	3.7609	7.5542	9.5269	38.7133	2.6598
Figure 3c	2	16,16	4.9984	7.5516	11.8834	51.0562	3.7920
Figure 3d	3	16,16,16	5.6034	7.5394	12.7895	58.1641	5.3340
Figure 3e	4	16,16,16,16	5.7891	7.4672	13.1481	60.6308	6.9986
Figure 3f	5	16,16,16,16,16	5.7363	7.2994	13.2229	60.5058	8.5886

**Table 2:** An objective evaluation of the NSST decomposition levels for Figure 4.

Figure	Levels (j)	$\mathrm{Directions}\;(2^{l})$	AG	H	SF	EI	RT
Figure 4a	/	/	2.3300	7.5341	10.3475	24.0514	0
Figure 4b	1	16	4.5223	7.2774	17.1468	42.1044	18.4116
Figure 4c	2	16,16	6.3055	7.2848	19.5768	61.0428	35.8895
Figure 4d	3	16, 16,16	6.8069	7.3293	20.1561	67.3551	53.7948
Figure 4e	4	16,16,16,16	6.9036	7.4187	20.2654	68.8586	75.4546
Figure 4f	5	16,16,16,16,16	6.8085	7.4351	20.1713	68.1701	86.2375

**Table 3:** An objective evaluation of the NSST shearing directions for Figure 6.

Figure	Levels (j)	$\mathrm{Directions}\;(2^{l})$	AG	H	SF	EI	RT
Figure 6a	3	16,16,16	5.6034	7.5394	12.7895	58.1641	5.3340
Figure 6b	4	4,4,4,4	**5.8223**	**7.4581**	**13.2668**	**61.2474**	**2.9424**
Figure 6c	4	4,4,8,8	5.8528	7.4573	13.2985	61.4832	3.5790
Figure 6d	4	8,8,4,4	5.782	7.4581	13.1731	60.7947	3.6289
Figure 6e	4	8,8,8,8	5.8122	7.4612	13.2097	61.0277	4.1941
Figure 6f	4	8,8,16,16	5.8356	7.4599	13.2460	61.2155	5.6572
Figure 6g	4	16,16,8,8	5.7650	7.4683	13.1010	60.4517	5.5436
Figure 6h	4	16,16,16,16	5.7891	7.4672	13.1481	60.6308	6.9986

**Table 4:** An objective evaluation of the NSST shearing directions for Figure 7.

Figure	Levels (j)	$\mathrm{Directions}\;(2^{l})$	AG	H	SF	EI	RT
Figure 7a	3	16,16,16	6.8069	7.3293	20.1561	67.3551	53.7948
Figure 7b	4	4,4,4,4	**7.3331**	**7.4109**	**21.0909**	**73.0702**	**22.6136**
Figure 7c	4	4,4,8,8	7.1990	7.4119	21.0370	72.0519	30.6810
Figure 7d	4	8,8,4,4	7.2375	7.4133	20.9472	71.9375	30.6267
Figure 7e	4	8,8,8,8	7.1001	7.4142	20.8958	70.8976	38.6901
Figure 7f	4	8,8,16,16	7.0193	7.4163	20.4215	70.2705	54.8658
Figure 7g	4	16,16,8,8	6.9897	7.4174	20.7423	69.5446	55.5662
Figure 7h	4	16,16,16,16	6.9036	7.4187	20.2654	68.8586	75.4546

**Table 5:** The average objective evaluation of the methods on the 30 chest X-ray images.

Figure	Levels (j)	$\mathrm{Directions}\;(2^{l})$	AG	H	SF	EI	RT
Figure 8a	3	16,16,16	8.3583	7.4553	20.4879	84.1216	31.3269
Figure 8b	4	4,4,4,4	**8.7476**	**7.4315**	**21.4071**	**88.3668**	**15.2641**
Figure 8c	4	4,4,8,8	8.6903	7.4313	21.2207	88.0425	19.6420
Figure 8d	4	8,8,4,4	8.6895	7.4350	21.2853	87.7092	20.5452
Figure 8e	4	8,8,8,8	8.6322	7.4351	21.1022	87.3851	24.9692
Figure 8f	4	8,8,16,16	8.5567	7.4352	20.8234	86.7944	33.6135
Figure 8g	4	16,16,8,8	8.5685	7.4401	20.9832	86.6336	35.4185
Figure 8h	4	16,16,16,16	8.4898	7.4401	20.7023	86.0090	42.0250

**Table 6:** Objective index analysis of methods for image enhancement.

Input	Index	Original	FLM	AGCWD	TSSR	LCM-CLAHE	Proposed
(1)	AG	1.0209	3.7850	1.4918	1.4474	2.7640	**5.8223**
	H	6.7705	**7.6702**	6.7923	7.0087	7.1667	7.4581
	SF	3.2987	10.0261	3.6482	3.8214	6.2908	**13.2668**
	EI	11.0071	38.4831	15.8314	15.4658	28.8997	**61.2474**
(2)	AG	2.3300	3.2646	2.7096	2.5508	4.3032	**7.3331**
	H	**7.5341**	6.6296	7.2817	6.2981	7.3906	7.4109
	SF	10.3475	20.3085	13.1887	12.1677	12.5993	**21.0909**
	EI	24.0514	31.7895	28.0340	26.3186	43.4980	**73.0702**
(3)	AG	2.7952	3.8904	3.0928	3.7314	3.9837	**6.3108**
	H	6.0726	6.2112	6.2114	5.1526	6.4169	**6.7970**
	SF	10.4788	16.4859	10.6596	13.6344	11.9184	**23.6223**
	EI	30.4747	39.5424	34.0402	40.6691	42.9675	**67.1452**
(4)	AG	1.3866	2.5508	1.5810	1.7410	2.5079	**4.1480**
	H	6.9763	5.9883	5.9370	6.5906	7.0725	**7.5378**
	SF	5.3728	12.9020	6.4922	7.5981	7.3069	**12.9766**
	EI	14.9979	25.8824	17.1513	18.8056	26.7857	**42.5292**
(5)	AG	2.3483	2.4361	2.6111	2.4574	3.1436	**4.5574**
	H	6.3407	5.3683	5.9630	4.0854	6.2908	**6.5791**
	SF	7.7697	9.0802	8.1054	10.0210	9.3806	**14.3243**
	EI	25.9900	26.2052	28.8516	27.4110	33.3082	**47.2777**

**Table 7:** Objective index analysis of the methods on the 30 COVID-19 X-ray images.

Input	Index	Original	FLM	AGCWD	TSSR	LCM-CLAHE	Proposed
30	AG	1.9192	3.6877	2.6340	2.6047	3.9789	**8.7476**
	H	7.2153	6.5179	7.3022	7.1615	7.4034	**7.4315**
	SF	6.4099	14.5301	8.4112	8.4708	9.7797	**21.4071**
	EI	19.6104	35.4322	26.8381	26.6882	40.3258	**88.3668**
	RT	0	4.3355	**0.0539**	0.2668	5.5797	14.8398

## Data Availability

Not applicable.
